# Acute Effects of Running on Shear Wave Elastography Measures of the Achilles Tendon and Calf Muscles in Professional Female Handball and Volleyball Players

**DOI:** 10.3390/diagnostics13182957

**Published:** 2023-09-15

**Authors:** Claudia Römer, Kirsten Legerlotz, Julia Czupajllo, Thomas Fischer, Bernd Wolfarth, Markus Herbert Lerchbaumer

**Affiliations:** 1 Department of Sports Medicine, Charité Universitätsmedizin Berlin, 10115 Berlin, Germany; 2 Movement Biomechanics, Institute of Sport Sciences, Humboldt-Universität zu Berlin, 10115 Berlin, Germany; 3 Department of Radiology, Charité Universitätsmedizin Berlin, 10115 Berlin, Germany

**Keywords:** shear wave elastography, ultrasound, Achilles tendon, professional athletes

## Abstract

Shear Wave Elastography (SWE) is currently used to detect tissue pathologies, i.e., tendinopathy. For preventive medicine, it is important to examine the sensitivity of SWE and to investigate how stiffness measures are affected by methodological variables. The aim of this study is to examine shear wave elastography (SWE) measures in order to compare the pre- and post-running values and to determine the correlation between the shear wave speed values (m/s). SWE examinations of the Achilles tendon (AT), soleus muscle (MS) and gastrocnemius muscle (MG)) were performed in 24 healthy professional female athletes. Measurements of the shear wave speed (m/s) were taken before and after incremental treadmill running until exhaustion. Correlations were investigated using the Pearson correlation coefficient and were examined for significance using the Student’s *t*-test. The pre- and post-exercise shear wave speed did not differ. The pre-exercise and post-exercise stiffness for MS (r = 0.613), MG (r = 0.609) and AT (r = 0.583) correlated strongly. The pre-exercise values and changes in stiffness showed a significant correlation (*p* < 0.001). In professional athletes, acute exercise induces different tissue stiffness changes in AT, MS and MG for each individual. Thus, exercise activity immediately prior to the SWE measurement needs to be factored in when evaluating tissue stiffness.

## 1. Introduction

Shear wave elastography is an upcoming technique for multiparametric ultrasound in musculoskeletal tissue. First, the application and the technical aspects of SWE will be outlined. Second, the state-of-the-art of SWE data are presented to understand the possibility of SWE in diagnosing pathologies, e.g., tendinopathies. However, these data are not generalizable because those examinations did not consider professional athletes. Third, the state-of-the-art of SWE examinations in professional sports are given to lead to the aim of the study.

### 1.1. Development of Ultrasound as Multiparametric Technique

As a result of ongoing technical improvements, ultrasound (US) has become more important in functional musculoskeletal (MSK) imaging. Moreover, applications such as shear wave elastography (SWE) or microflow imaging add a quantitative evaluation of muscle or tendon tissue, and have therefore introduced multiparametric US (mpUS) into MSK imaging [1,2,3]. SWE has been widely used in the medical field, particularly when diagnostics with computer tomography and ionizing radiation is inappropriate, such as in very young patients. It can also be a complementary diagnostic technique in distinguishing inflammatory or fibrotic tissue [4]. SWE provides quantitative data, where contrast enhanced ultrasound (CEUS) is limited to qualitative data [4] and shows great potential for monitoring and evaluating progress and the effectiveness of therapy or rehabilitation [4].

However, SWE needs to be examined further as SWE data taken before and after acute exercise are missing. As tendon stiffness values may vary in relation to exercise activity, it is necessary to examine the acute effect of exercise on the musculoskeletal tissue measured using SWE in professional athletes. This will foster our understanding of tissue stiffness changes in professional athletes, which is needed to be able to develop diagnostic approaches in preventive medicine. As female professional athletes are still under-represented in research [5], this study focused on female professional athletes with comparable types of sports, training hours per week, ages and BMIs.

### 1.2. Shear Wave Elastography Application

Ultrasound is the fastest method for assessing tendon and muscle pain. Particularly with the rising availability of point-of-care ultrasound tools, it is a valuable complementary tool alongside the clinical examination for musculoskeletal injuries. In contrast to strain elastography, shear wave elastography showed good intra- and inter-observer reliability in musculoskeletal tissue [6,7], is easy applicable and the color-coded map allows for fast tissue stiffness interpretation. SWE is based on measurements of shear wave speed (SWS). The propagation speed of the shear waves in the tissue can be detected directly as a shear wave speed in the unit of m/s, with a high speed for stiff and low speed for soft tissue [8,9], and can thus be used as a surrogate for tissue stiffness in the form of a quantitative parameter using the derivation of Young’s modulus [10]. In addition, it can be safely applied to investigate both healthy and injured tendons and muscles, as no movement of the joint is required to detect elasticity. This allows for the easy and fast assessment of tissue stiffness in both muscles and tendons.

### 1.3. Shear Wave Elastography in Musculoskeletal Tissue

SWE has shown good specificity in diagnosing tendinopathy and discriminates between intra-individual pathological tendon changes and contralateral healthy tendons [7,11,12]. Dirrichs et al. examined the application of SWE for the diagnosis of tendinopathies of the Achilles and patellar tendon [13]. Indeed, SWE, compared to B-mode and power Doppler, showed a significantly lower rate of false-positive results and correlated stronger with the clinical symptoms [13]. SWE has shown the potential to identify inflammatory tissue, which can be detected by measuring decreased stiffness values in the tendon tissue [4,7]. As the shear wave velocity can be determined for small regions, SWE offers the possibility to compare specific areas within the tissue. This could be useful, for example, in the diagnosis of local pathological tissue changes such as tendinopathies or muscle fibre ruptures.

### 1.4. Shear Wave Elastography in Professional Sports

Tendinopathy is a serious condition in professional sports and is highly correlated with a reduction in training, and can also be career ending [14]. SWE can be helpful for detecting early tissue stiffness changes, which can be a sign of the beginning stage of tendinopathy [13,15].

It is already known that long-term exercise activity in general, and endurance training in particular, affects the shear wave speed (SWS) [16,17]. Furthermore, SWE can be used to monitor the training effects of resistance training in muscle vastus lateralis [18,19] and the effects of overuse after extreme endurance running in quadriceps running, which is a sign of inflammation [20]. SWE can be applied in both musculoskeletal pathologies and in healthy athletes to examine tendon and muscle tissue. An examination of the Achilles tendon showed higher SWE values in recreationally active subjects in comparison to non-active subjects [17]. In addition, SWE has been shown to be sensitive to acute changes in muscle stiffness during muscle contraction [10,21]. SWE is a further diagnostic method to approach tendon and muscle stiffness in semi-professional and professional athletes [17,22].

Although there is data for diagnosing tendinopathy of the Achilles tendon and patellar tendon using SWE, studies examining the influence of an acute exercise—e.g., a training session in professional athletes—are missing. This information is necessary for discovering whether SWE measurements are suitable for determining musculoskeletal tissue stiffness for professional athletes on an active training day.

The aim of this study is to evaluate tendon and muscle stiffness before and after a standardized treadmill exercise in professional female athletes to investigate the influence of acute training exposure on Achilles tendon and calf muscle stiffness and SWE measurement. The primary objective is to examine and compare the pre- and post-treadmill running SWS values (m/s) of the Achilles tendon, soleus muscle and gastrocnemius muscle and to determine the correlation. The secondary purpose of this study is to investigate the differences between the Achilles tendon and calf muscle stiffness values (m/s) between professional female handball players and volleyball players. This study focuses on the Achilles tendon and calf muscles as Achilles tendinopathy is a common injury in professional athletes. Chronic tendinopathy leads to a high absence of training and can be career-ending [23]. Calf muscles were examined to understand the stiffness changes in the muscle-tendon-unit.

## 2. Methods

### 2.1. Participants

The study included 24 healthy professional female volleyball and handball athletes, who were examined from June until August 2021. Inclusion criteria were: (I) age ≥ 18 years; (II) healthy female professional athletes (>10 h training per week); (III) without any acute (>6 months) musculoskeletal, rheumatic or vascular comorbidities and no previous injuries of Achilles tendon, soleus muscle, gastrocnemius muscle; and (IV) written informed consent to participate in the study. The following exclusion criteria were applied: tendon thickening, tendon neovascularization and hypoechogenity. The study was conducted in accordance with the Declaration of Helsinki and with the approval of the local ethics committee of Charité University Medicine Berlin (EA2/162/19).

**Figure 1:**
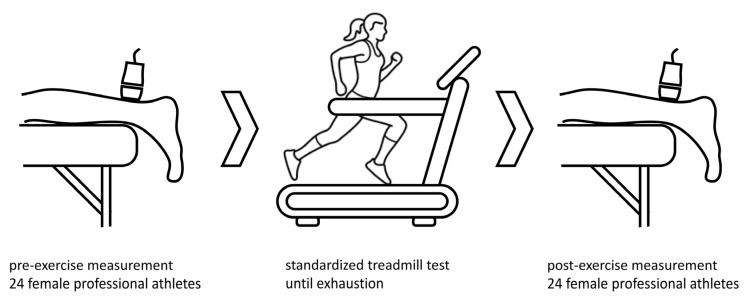
Measurements of shear wave speed (m/s) were taken before and after a standardized treadmill test until exhaustion to determine stiffness and shear wave speed of Achilles tendon and calf muscles after an acute exercise. Icon source: O. Panasovskyi, thenounproject.com (accessed on 27 August 2023).

Baseline patient characteristics were acquired at the time of the first examination. On the day of the measurements and treadmill exercise test, no training was performed before testing. SWE investigation was performed on twenty-four athletes before and after a standardized running exercise, as shown in Figure 1. The running protocol was performed on a treadmill (T170 h/p/cosmos) with 1% incline. The treadmill velocity was 6 km/h at the start, and was increased by 2 km/h every three minutes. The protocol lasted 18 to 21 min until individual exhaustion. Maximal treadmill speed was 12 to 14 km/h. After the treadmill exercise, SWE measurements were performed again to examine the stiffness of the Achilles tendon, soleus muscle and gastrocnemius muscle. All measurements were jointly performed by a trained sonographer and a highly experienced radiologist. SWE measurements were performed as part of a pre-season examination, including complete physical examination, ECG, lung function test, laboratory diagnostics and lactate performance diagnostics. All athletes were approved for next season participation.

The median age of the athletes and the median body mass index (BMI) are shown in Table 1. One athlete had been diagnosed with hypothyreosis, while no other diseases, such as diabetes, fatigue, hyperlipidemia, rheumatic diseases or malposition of lower limb joints, were reported. Overall, eleven athletes reported a rupture of lower ligaments (ligament rupture of the ankle [*n* = 8]), ligament rupture knee [*n* = 7]). None reported a tendon rupture. Medication currently being taken were L-Thyroxin (*n* = 1) and hormonal contraceptives (oral [*n* = 4], intrauterine device [*n* = 2]).

Thirteen athletes were members of the volleyball national team and eleven of the German handball league. Overall, 84% of all of the athletes trained more than 10 h per week; 16% trained 5–10 h per week.

### 2.2. Shear Wave Elastography Protocol

All SWE measurements of the subjects were performed on the same day, applying a standardized protocol. For assessment of the Achilles tendon mid portion stiffness, participants were examined in the prone position with both feet hanging in relaxed position over the examination couch. Leg position was maintained for the measurement of the soleus muscle insertion (MS) and lateral part of the gastrocnemius muscle (MG). Prior to SWE, gray-scale B-mode US was performed in the transverse and longitudinal planes for adequate assessment of the tendon, muscle and probe position. After performing B-mode US to detect the adequate measurement position, this area was marked with a permanent pencil to ensure the same measurement point for the pre- and post-exercise SWE. All examinations were performed using an US system with a 4–10 MHz multifrequency linear array transducer and a center frequency of 7 Mhz (Acuson Sequoia, Siemens Healthineers, Erlangen, Germany). The SWE software (Virtual Touch™) allows for real-time measurement using Acoustic Radiation Force Impulse (ARFI) imaging technology for the quantitative evaluation of shear wave speed.

**Figure 2:**
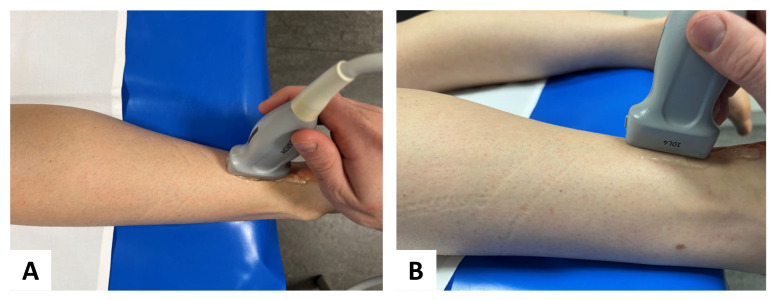
Probe placement in standardized position: for SWE, a multifrequency linear transducer was placed in longitudinal plane for accurate assessment of the Achilles tendon (**A**) aerial perspective and (**B**) lateral perspective.

US examinations were performed in the longitudinal plane relaxed positions (Figure 2) to depict each tendon and the area of interest in one single image. Using the respective 2D SWE approach, the examiner acquired four US images of each tendon and muscle of both legs with consecutive SWE measurements using a 3-mm circular region of interest (ROI) placed in the center of each target tendon, avoiding areas of visible artifacts. Thus, representative tendon stiffness is given as the median of 12 measurements and the corresponding interquartile range. Before ROI placement, SWS was depicted through color-coded SWE mapping. The standardized penetration depth was adapted to each participant for optimal visualization of the tendon and correct SWE measurement. US-gain was not changed to avoid potential effects on the SWE measurements.

### 2.3. Statistical Analysis

To determine the normality of the continuous variables, the Kolmogorov-Smirnov test was applied. Variables that were not normally distributed are reported as median and interquartile range (IQR). Categorical variables were analyzed using the Student’s *t*-test for the secondary study objective and presented as proportions, both as absolute values and percentages. Correlations were investigated using the Pearson correlation coefficient and examined for significance using Student’s *t*-test for the primary study objective. A significance level of *p* < 0.001 was determined and all statistical analysis was performed using both the SPSS software (IBM Corp., released 2019. IBM SPSS Statistics for Windows, Version 26.0. Armonk, NY, USA: IBM Corp.) and Matlab (MATLAB and Statistics Toolbox Release 2022b, The MathWorks, Inc., Natick, MA, USA).

## 3. Results

### Results of SWE Examination

**Figure 3:**
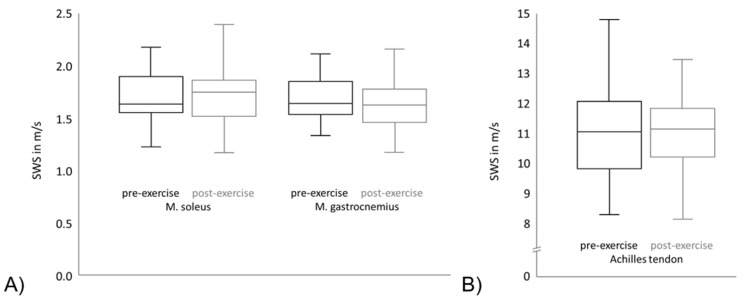
Shear wave speed (SWS) of pre- and post- exercise measurements for (**A**) the Achilles tendon and (**B**) the soleus and gastrocnemius muscle.

As visualized in Figure 3, no significant change in the median SWS could be observed between the intra-individual pre- and post-exercise measurements in the Achilles tendon, the soleus or gastrocnemius muscle (values presented in Table 2). Figure 3A shows the SWS of the soleus muscle and gastrocnemius muscle. The black boxplots point out the pre-exercise values of the SWS of all subjects and the grey boxplots show the post-exercise values. It can be seen that the means and variation of the SWS is comparable between different muscles. Furthermore, regarding the SWS means and variation of each muscle, the pre-exercise and post-exercise values are comparable. Figure 3B shows the SWS of the Achilles tendon, which is significantly higher in comparison to the SWS of both muscles. As in Figure 3A, the black boxplot points out the pre-exercise SWS values and the grey boxplot shows the post-exercise SWS values. The means and variation are comparable between the pre-exercise and post-exercise values in the Achilles tendon.

**Figure 4:**
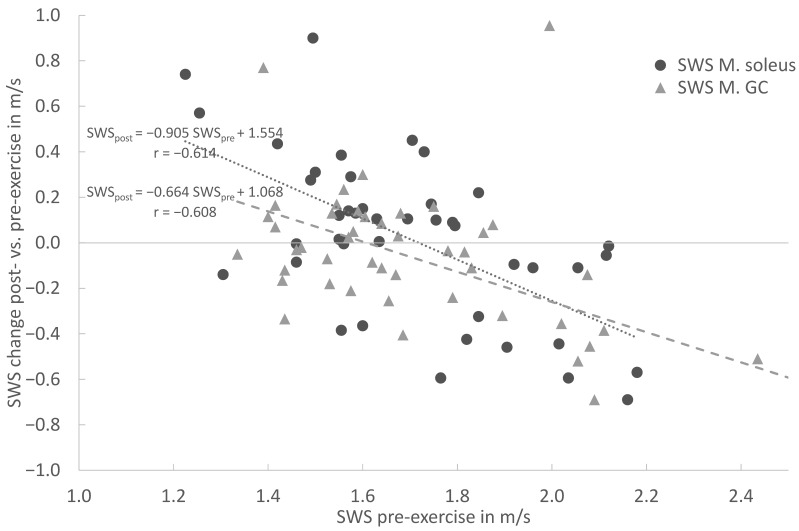
Linear regression of shear wave speed (SWS) change post- vs. pre-exercise measurements in comparison with pre-exercise SWS (x = SWS in m/s; y = delta (SWS_post_ − SWS_pre_) for Soleus muscle (circle) and Gastrocnemius muscle (triangle).

**Figure 5:**
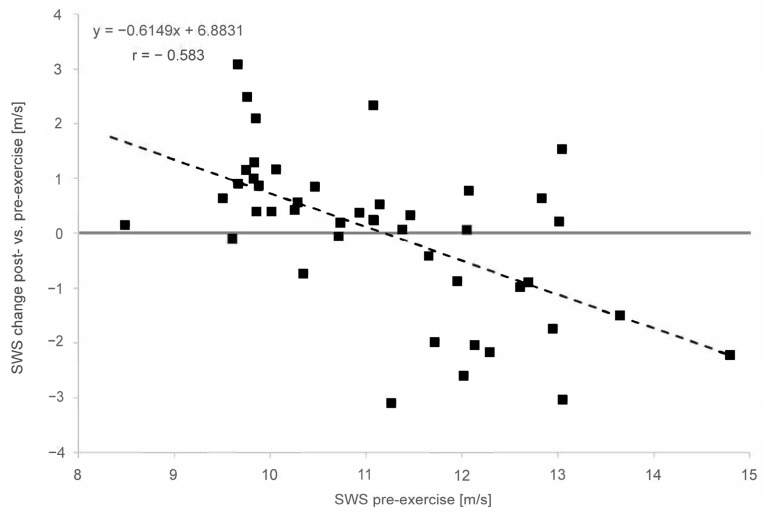
Linear regression of shear wave speed (SWS) change post- vs. pre-exercise measurements in comparison with pre-exercise (x = SWS in m/s; y = delta (SWS_post_ − SWS_pre_) for Achilles tendon. The solid line is the vertical axis value zero (no SWS change post- vs. pre-exercise. The dashed line indicates linear regression.

In the overall cohort, the baseline SWE assessment demonstrated a moderate to strong correlation between the pre-exercise and post-exercise stiffness for the MS (r = 0.613), MG (r = 0.609) and AT (r = 0.583). In both muscles and the tendon, the linear regression shows an increase in stiffness for lower pre-exercise values and decreasing stiffness for higher pre-exercise values (Figure 4 and Figure 5). This covariance between the pre-exercise values and changes in stiffness is highly significant (*p* < 0.001 for MS, MG and AT, cf. Table 2).

The linear regression (Figure 4) shows an increase in stiffness for lower pre-exercise values and decreasing stiffness for higher pre-exercise values for the Soleus and Gastrocnemius muscles. This covariance between the pre-exercise values and changes in stiffness is highly significant (*p* < 0.001 for MS and MG). For both the MS and MG, the respective regression models are given in Figure 4. In particular, for the MS (circle), the slope of −0.9045 indicates an almost complete compensation of the pre-exercise SWS towards the post-exercise SWS. Both models intersect with the horizontal zero SWS change, straight between the 1.6 and 1.7 m/s pre-exercise SWS, and are similar to the distribution’s mean values.

The linear regression (Figure 5) shows an increase in stiffness for lower pre-exercise values and decreasing stiffness for higher pre-exercise values for the Achilles tendon. This covariance between the pre-exercise values and changes in stiffness is highly significant (*p* < 0.001 for AT). The regression model’s slope and intercept values for the AT are also given in Figure 5. Again, the model’s intercept with the horizontal zero SWS change straight at 11.2 m/s is similar to the SWS pre-exercise median value of 11.08 m/s for the AT.

**Figure 6:**
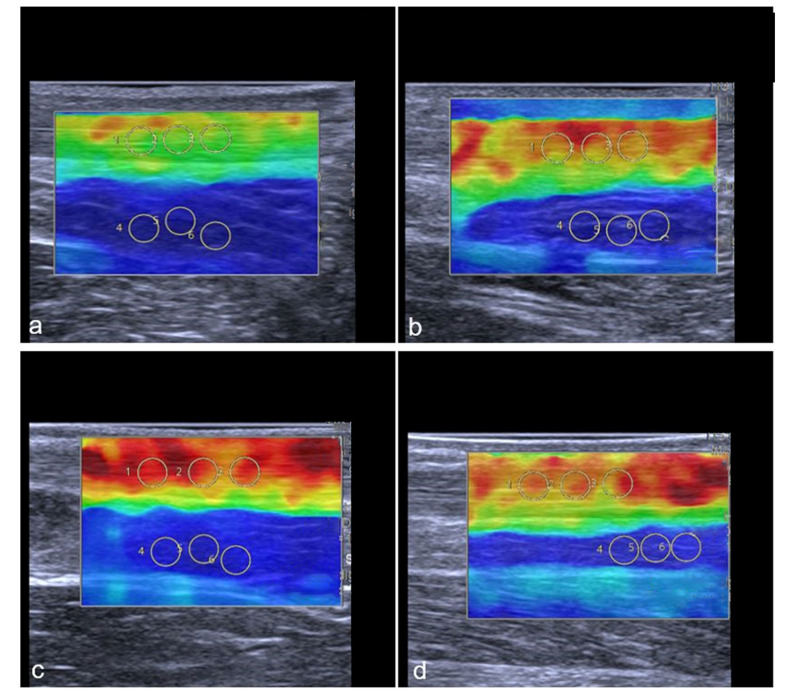
Representative SWE images of two professional female athletes prior and after standardized treadmill. Color-coded map of Achilles tendon is shown in (**a**–**d**). Green corresponds with softer tendon tissue and lower SWS values. Subfigures (**a**–**d**) show the same female athlete, (**a**,**c**) demonstrate pre-exercise images. (**b**,**d**) demonstrate post-exercise images. The color-coded SWE maps in Figure 6 present the AT stiffness of two athletes. In the 2D-SWE color maps, red is coded as hard tissue and blue as soft. The overall SWS was calculated using four images with three regions per structure, given an overall number of 12 measurements per tendon or muscle. In this figure, a female athlete with lower baseline SWS (**a**) showed an increase in stiffness after exercise (**b**), while another athlete with higher baseline stiffness (**c**) showed a slight reduction in SWS (**d**) (Gastrocnemius muscle not shown in this images).

Representative images of the change in the tendon stiffness are shown in Figure 6.

There was no difference in the tendon and muscle SWS between the type of sports (volleyball [AT 11.11 m/s; MS 1.75 m/s; MGC 1.70 m/s] and handball [AT 11.62 m/s; MS 1.8 m/s; MGC 1.76 m/s]; *p* > 0.05).

## 4. Discussion

Shear wave elastography is described as an additional ultrasound method for detecting tendon and muscle pathologies and can be a valuable diagnostic tool in prevention and rehabilitation for professional athletes. There is a lack of evidence as to whether SWE is sensitive to tissue changes in the lower limb after a single training session in professional athletes. This is important as examinations of professional athletes are regularly scheduled after training sessions. The aim of this study was therefore to examine the impact of acute exercise on the calf muscles and Achilles tendon, measured through SWE. While the median shear wave speed did not differ between the pre- and post-exercise SWS values, we detected an increase in stiffness for lower pre-exercise values and decreasing stiffness for higher pre-exercise values in the professional female athletes.

### 4.1. Shear Wave Elastography of Achilles Tendon

Exercise activity prior to the measurement can affect the results, as indicated by the increase in stiffness for lower pre-exercise SWS values and the decreasing stiffness for higher pre-exercise values in healthy tendons. This implies an individual response of healthy tendons and muscles to a mean optimal stiffness after a short training session in professional athletes. These individual post-exercise changes should be considered when diagnosing tendon injuries. Transient changes in stiffness after heel drop exercises have been reported; however, only in non-professional participants [24]. Decreased tendon stiffness was detected after long distance running [25]. Risch et al. reported a higher intra-tendinous blood flow in the AT shortly after a treadmill running exercise in recreational and semiprofessional athletes in some participants, while the other ATs were unaffected [26]. In this study, stiffer ATs in particular showed decreased stiffness values after acute exercise, which could be a sign of higher intra-tendinous blood flow. The missing change of tendon stiffness in our cohort may be driven by a professional sports collective with a general high amount of training hours and ongoing stretching exercises combined with physiotherapy care. This study did not detect a difference in the stiffness between the volleyball and handball players. This may be due to the similar training load and similar kinds of exercise activities, such as running and jumping.

Professional female athletes seem to have a common AT stiffness after acute exercise between 11 m/s and 12 m/s. Although cut-off values are only useful for one ultrasound system, the general knowledge of changes in tendon stiffness regarding professional athletes is highly important for future research and potential prospective studies. Reference values and stiffness changes shortly after exercise also need to be considered while using SWE in preventive and rehabilitation medicine, as well as the training load [22].

### 4.2. Shear Wave Elastography of Soleus und Gastrocnemius Muscle

SWE is also an appropriate technique for assessing muscle stiffness [2] and shows good inter-operator results [27]. Changes in muscle stiffness can be detected by SWE even when muscle tension is monitored [10]. This study found an increase in stiffness for lower pre-exercise SWS values and decreasing stiffness for higher pre-exercise values in healthy soleus and gastrocnemius muscles. The most important factor for obtaining the correct measurement is controlling for the exact joint angle, as SWE is sensitive to different joint angles and passive flexion [28].

### 4.3. The Potential of Shear Wave Elastography to Detect Inflammatory Tissue

For assessing SWE measurements, it is necessary to adhere to a standardized measurement protocol; joints, tendons and muscles need to be in a relaxed position and transducer positioning should be performed without pressure as the joint position, contraction and pressure do have an impact on SWS [29]. Regarding these influences, SWE is a reliable technique for assessing muscle and tendon stiffness [30]. In addition to musculoskeletal tissue, SWE has shown encouraging results in inflammatory tissue. Mazza et al. showed a good correlation between magnetic resonance enterography (MRE) and elastography in fibrotic intestine changes and strictures [31]. They proposed a predictive conclusion for pending surgery or hospitalization [31]. Furthermore, SWE seems to have a good predictive value for detecting inflammatory tissue, which may offer a significant diagnostic benefit as MRE is a time-consuming and scarce examination modality. Shear wave elastography can be applied directly in the emergency room or on the ward [31]. In addition, SWE shows a good reproducibility in further tissue pathologies. In patients with chronic liver disease, SWE showed a correlation with liver tissue changes and fibrosis and reduces the necessity of liver biopsies [32], which is beneficial as these patients often present coagulation disorders. Complication rates may be reduced by using a combined approach with CEUS and SWE. SWE also demonstrated higher stiffness in the median nerves in patients with carpal tunnel syndrome [33], which reveals its potential use for musculoskeletal tissue. SWE is based on measurements of SWS and showed potential value in the diagnosis of injuries, and especially in discriminating tendinopathies with intra-individual changes compared to contralateral healthy tendons [7,11,12]. These results point out the potential of SWE, not only in healthy individuals, but also in detecting therapy progress. SWE can be used to detect differences in physically active and non-active individuals [17]. When assessing stiffness values measured by SWE, the effects of sex need to be considered [6,34].

#### Perspective Section

Overall, several studies have shown that SWE is a suitable diagnostic tool for assessing the stiffness of tendons and muscles, with potential application in rehabilitation and preventive medicine [35,36,37]. Individual regular assessment of tissue health using SWE in professional sports could detect decreasing stiffness of the Achilles tendon, which may be associated with an increased risk of injury or the beginning of inflammation [7]. Thus, intra-individual changes in tendon stiffness measured by SWE provide additional information and could be implemented as a screening tool during long-term training plans or high-volume training camps to prevent injuries. Furthermore, Dirrichs et al. concluded that SWE provided good results for differentiating between subjects with painful tendinopathies and asymptomatic, healthy control subjects [13]. The results were consistent with the VISA-A Score in subjects with Achilles tendinopathy [38].

Standardized SWE measurement protocols with precisely localized transducer positions and exactly defined positions of the test subjects during the measurement would further increase the comparability of SWE data in MSK studies [39,40]. More precise and comparable research protocols in MSK-SWE could offer a great benefit in sports medicine as well as in rehabilitation and preventive medicine [12,13,41,42,43,44].

### 4.4. Limitations

Cycle phases and oral contraception pill intake were not considered. Active muscle contraction was described to be higher during menstruation, while resting muscle stiffness was not influenced by cycle phases [45]. Oral contraceptives may affect muscle regeneration [46], which needs to be investigated in further studies. Female professional athletes were examined because there is a research gap for this clientele, although high injuries rate are described in the literature [47].

Furthermore, B-mode power Doppler was not applied in the post-exercise measurements to evaluate a higher or lower blood flow. SWE should be applied in future studies as a multiparametric US for a better understanding of stiffness changes measured by SWE. Pathologies in the musculoskeletal tissue should be assessed through a combination of B-mode power Doppler and SWE. Further studies should also examine male athletes and older athletes and the impact of daily activity before SWE measurements in healthy volunteers. US and SWE should be applied by a highly experienced sonographer in specialized centers, including a focus on musculoskeletal tissue imaging.

To summarize, in professional athletes, acute exercise induces individually different tissue stiffness changes in the AT, MS and MG. Thus, exercise activity immediately prior to the SWE measurement needs to be factored in when evaluating tissue stiffness.

## 5. Conclusions

Shear wave elastography may be applied to point out pathological changes, as changes in the stiffness values correlated with restricted mobility or pain, especially in tendinopathy and muscle inflammation. Regular longitudinal tendon monitoring during the therapy process showed beneficial results for SWE. For professional athletes, easy access diagnostics are necessary to monitor muscle and tendon stiffness to detect the early stages of injuries and to develop preventive therapy algorithms to avoid difficult-to-treat tendon and muscle injuries. Regular longitudinal SWE measurements in combination with a standardized sports medical examination once a year and pre- and post-season SWE measurements may help trainers and doctors to adapt training protocols for the individual athlete to prevent chronic tendon and muscle injuries. This is essential in preventive and rehabilitation medicine for professional athletes and adolescent athletes as lateral imbalances are a risk factor for future injuries and can be detected using SWE [48]. In this context, further studies are still necessary, as well as further standardization of the measurement method of SWE to improve the consistency of SWE measurements and enhance the comparability of SWE studies.

Our study revealed that in professional athletes, acute exercise induces different tissue stiffness changes for each individual, which needs to be considered when applying SWE for diagnostic purposes. While considering these findings in the clinical context, regular screening methods should be complemented by shear wave elastography for sport-specific exposed tendons and muscles as this may help physicians to point out the early signs of injury.

## Figures and Tables

**Table 1:** Median age and BMI are shown. VB: volleyball, HB: handball.

	Female Athletes (*n* = 24)	
Age	21	18–26
BMI	22.47	19.60–27.82
VB	13	
HB	11	

**Table 2:** Covariance between pre-exercise SWS and the SWS change towards post-exercise.

Estimation	MS	*p*-Value	MG	*p*-Value	AT	*p*-Value
Intercept	1.5540	<0.001	1.0682	<0.001	6.8831	<0.001
SWS pre-exercise (m/s)	−0.9045	<0.001	−0.6644	<0.001	−0.6149	<0.001

## Data Availability

The data presented in this study are available on request from the corresponding author. The data are not publicly available due to data privacy regulations.

## Abbreviations

AT	Achilles tendon
MRI	magnetic resonance imaging
MS	musculus soleus
MG	musculus gastrocnemius
SWE	shear wave elastography
SWS	shear wave speed
US	ultrasound
